# Thermal Mitigation Behaviors of Captive Blue Peafowls and Visitors’ Outdoor Thermal Comfort: A Case Study at Jinan Zoo, China

**DOI:** 10.3390/ani15050700

**Published:** 2025-02-27

**Authors:** Zhiqiang Zhou, Ran Jiao, Huijian Hu, Tauheed Ullah Khan

**Affiliations:** 1 College of Architecture and Urban Planning, Guangzhou University, Guangzhou 510006, China; 2 Guangzhou Municipal Group Design Institute Co., Ltd., Guangzhou 510095, China; 21014085038@stu.hqu.edu.cn; 3 Guangdong Key Laboratory of Animal Conservation and Resource Utilization, Institute of Zoology, Guangdong Academy of Sciences, Guangzhou 510260, China; 13922339577@139.com (H.H.); eco.tauheed@hotmail.com (T.U.K.)

**Keywords:** zoo, blue peafowl, visitors, outdoor thermal environment, management, animal welfare

## Abstract

Zoos play dual roles in wildlife conservation and in providing recreational experiences for visitors in urban green spaces. However, the impacts of thermal environments on both visitor comfort and captive animal welfare remain unexplored, which is an important aspect to address for improving overall zoo management. This study investigated thermal conditions at Jinan Zoo, China, over 20 summer days. Questionnaires were used to collect visitor thermal comfort and viewing satisfaction, while the thermal mitigation behaviors of 70 blue peafowls were recorded under various thermal conditions on-site. The findings showed that the wet-bulb globe temperature (WBGT) neutral range for visitors was 20.1–24.4 °C, with a significant drop in visitor numbers when WBGT exceeded 35.5 °C. Visitors with higher animal viewing satisfaction (aVSV) scores were more heat tolerant. The blue peafowls reduced their activity levels and displayed feather-spreading and gular flutter at WBGT levels of 26.4–30.4 °C, especially during peak visitor hours. Our study also showed that visitor thermal sensation was most affected by radiation, whereas blue peafowl heat stress was likely influenced by air temperature, followed by humidity and radiation. These findings offer practical insights for designing zoo enclosures and visitor areas to improve comfort and animal welfare in hot weather.

## Simple Summary

This study investigated the thermal adaptation behaviors of blue peafowls and the thermal comfort characteristics of zoo visitors during summer heat at Jinan Zoo, China. Visitor numbers remained relatively stable in thermal conditions exceeding the neutral range. However, a marked decline was observed when temperatures reached excessive heat levels. The blue peafowls alleviated heat stress through behaviors such as feather-spreading, panting, and reduced activity. During peak visitor hours, the frequency of these heat-relief behaviors remained elevated. Visitors with higher viewing satisfaction had a broader tolerance for thermal environments. Visitors were most affected by direct sunlight, while the peafowls were possibly most influenced by air temperature.

## 1. Introduction

Urban zoos are an integral part of green spaces and public facilities, making important contributions to the urban living environment [1,2]. As venues for public engagement with nature, they play a critical role in recreation, ecological education, and conservation. In recent decades, animal welfare has become a prominent social issue, attracting growing public attention [3,4,5,6,7] and emphasizing the need to improve animal well-being. Thermal comfort is a key aspect of animal welfare. However, rapid urbanization and global warming have not only affected the lives of urban residents but also posed challenges to zoo animal’s habitats and health. Zoo animals, particularly during summer heatwaves, face risks of heat stress, which can disturb their behaviors and well-being. Zoos are considered ideal sites for studying the interplay between animal welfare and human comfort under extreme climatic conditions. Previous studies have primarily focused on human thermal comfort [8,9,10,11,12,13,14,15], leaving zoo-specific thermal conditions unexplored.

There is limited literature on animal heat mitigation strategies, and these few studies mainly focus on mammals [16,17,18,19,20]. However, such studies rarely considered visitors’ thermal comfort alongside animal welfare. In addition to mammals, some studies have examined behavioral thermoregulation in wild birds under thermal stress. For example, Marder et al. [21] found that pigeons primarily dissipate heat through skin evaporation in high-temperature environments. According to Ryan et al. [22], when the environmental temperature exceeds the South African nightjar’s body temperature, its evaporative water loss rapidly increases to maintain thermal balance. Birds often employ strategies such as seeking shade (SS), rapid breathing, gular flutter (GF), and feather spreading (FS) to mitigate heat stress [23,24,25,26,27]. However, these heat mitigation behaviors in captive zoo birds remain poorly characterized.

Existing research indicates that due to the unique psychological expectations and activity patterns of outdoor visitors, their thermal comfort experiences differ from those of the general population [28,29,30,31,32]. Both the visitors and the animals are subject to the same thermal environment, yet it remains unclear whether there are differences in the thermal comfort needs of visitors and animals, and whether the animals’ thermal mitigation behaviors might affect the visitors’ experience, thereby influencing their thermal mitigations. These questions have not been explored in previous research.

An ideal zoo environment should ensure both animal welfare and visitor comfort. Positive visitor experiences also improve zoo sustainability efforts [33]. Therefore, studying the zoo’s thermal environment by integrating the thermal comfort and viewing experience of visitors with the thermal mitigation behaviors of animals can provide scientific guidance for zoo design and management. Thus, understanding how thermal conditions influence both animals and visitors is vital for improving zoo designs and management. This study focuses on the blue peafowl (*Pavo cristatus*), a representative zoo species known for its striking appearance, global popularity, and frequent presence in zoos, yet often overlooked in heat mitigation research.

This study used the peafowl enclosure in Jinan Zoo, China, as a case study to explore the thermal comfort of visitors and the thermal mitigation behaviors of animals under summer conditions. It quantitatively analyzes the impact of visitor satisfaction on their thermal perception and the correlation between viewing satisfaction and the thermal mitigation behaviors of the peafowls. Additionally, the study investigates how the thermal environment and other factors correlate with both blue peafowls’ heat mitigation behaviors and visitors’ thermal perceptions. By focusing on these interrelationships, the research provides actionable guidance for zoo design and management.

## 2. Materials and Methods

### 2.1. Study Site

**Figure 1:**
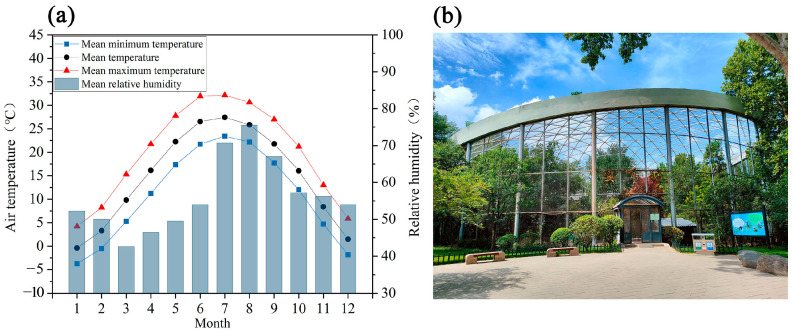
(**a**) Monthly ranges of temperature and relative humidity in Jinan (2000–2022). Source: China Meteorological Data Sharing Service System. (**b**) The Blue Peafowl enclosure facility at Jinan Zoo.

This study was conducted in Jinan, a city in northern China. The city experiences hot summers with high humidity and intense solar radiation, making summer heat a significant urban safety concern (Figure 1a). The research was carried out over a 20 day period, from 29 July to 17 August 2024, with daily observations conducted from 08:00 to 17:30 h. The research site was the blue peafowl (*Pavo cristatus*) enclosure at Jinan Zoo. The enclosure is a circular aviary covering approximately 2000 m^2^. It is enclosed by fine metal mesh, covered with a roof (Figure 1b).

**Figure 2:**
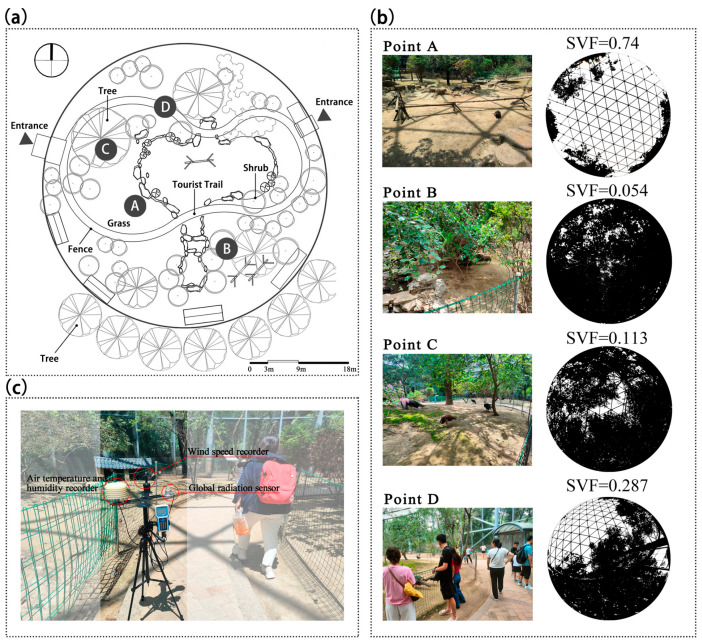
(**a**) Floor plan of the blue peafowl enclosure. A, B, C, and D were the observation points. (**b**) Photographs and Sky View Factors (SVFs) scores of the observation points. (**c**) Meteorological station instruments were used during the surveys to collect the required data.

Inside the enclosure, a circular visitor pathway provides a viewing route (Figure 2a). The metal mesh roof allows sunlight to penetrate, while various trees and shrubs having different heights are planted inside and around the enclosure to provide shade for visitors and the peafowls. The blue peafowls live within the enclosure, where the spacious environment allows them to move freely with minimal disturbance from zoo visitors.

### 2.2. Study Subjects: Zoo Visitors and Blue Peafowls

This study examined the thermal comfort of visitors in the zoo’s blue peafowl enclosure and the thermal mitigation behaviors of blue peafowls. Most visitors were residents of the Jinan and nearby cities, with a smaller proportion coming from more distant locations. The visitor group included children, adults, and elderly individuals.

The blue peafowl belongs to the class Aves, order Galliformes, family Phasianidae, and genus Pavo [34]. The species is widely distributed across South Asia, particularly in India and Sri Lanka [35]. Known for the male’s ornate tail feathers and vibrant plumage, the blue peafowl is a popular exhibit in zoos worldwide and a favorite among visitors. Adapted to high-temperature environments, blue peafowls employ a combination of behavioral and physiological strategies to manage heat stress. These include seeking shade to reduce sun exposure, spreading feathers to increase surface area for heat dissipation, and rapid breathing to enhance evaporative cooling [36]. The study group consisted of 70 locally bred mature blue peafowls in China, aged from 3 to 8 years. The group consisted of 36 males and 34 females.

### 2.3. Behavioral Observation

A digital camera (ZV-E10, Sony Corporation, Tokyo, Japan) was used to record the number of visitors in the peafowl enclosure. During the observation period, photographs of pedestrians along the circular pathway were taken every 15 min. To comprehensively reflect visitor numbers, 3–4 photos were taken along the circular pathway each time, depending on the number and distribution of visitors. To avoid duplications, individuals appearing in multiple photographs during a single observation session were counted only once.

The thermal mitigation behaviors of blue peafowls were recorded using a digital camera. Specific behaviors, included dynamic activities (DA), shade-seeking (SS), feather-spreading (FS), and gular flutter (GF) (Table 1). During the observation period, researchers walked along the circular pathway of the enclosure, recording multiple videos every 15 min to document the peafowls’ thermal mitigation behaviors. For each session, 3–4 videos were recorded along the pathway, depending on whether the peafowls were gathered or dispersed. Each recording session produced a 1 min video.

Since FS and GF are not continuous behaviors, the presence of these activities within a 1 min video was regarded as evidence that the bird exhibited dynamic activity or engaged in thermal mitigation. If these behaviors were absent, the bird was classified as static or as not having exhibited these specific thermal mitigation behaviors. Additionally, if a blue peafowl spent more than 30 s in the shade during a 1 min recording, it was categorized as staying in the shade; otherwise, it was recorded as staying in the sun. The same blue peafowl may appear in multiple videos within a single observation session, and to avoid duplicate counts, the peafowl’s location and surrounding facilities in the video were used as references for exclusion. Throughout the observations, the researchers remained on the visitor pathways to avoid disturbing the animals.

### 2.4. Thermal Environment Monitoring and Meteorological Indices

Mobile weather stations were used to monitor the thermal environment of the peafowl enclosure. The stations were equipped with sensors for measuring air temperature and humidity, an anemometer, and a global radiation sensor. The specifications of the meteorological instruments are listed in Table 2. Weather stations were deployed at four measurement points (Figure 2).

Measurement points A, B, and C were located in the peafowl activity area, with sensors positioned 0.4–0.6 m above the ground. Point D was placed along the visitor pathway, with sensors positioned 1–1.2 m above the ground. Point A was an open area in the enclosure with no tall trees or shrubs nearby, receiving direct sunlight for most of the day on clear days. Point B was in a shaded area with a combination of trees and shrubs. Point C was shaded by a tree. The sky view factor (SVF) scores for the four points were 0.74, 0.054, 0.113, and 0.287, respectively (Figure 2b).

Since the shaded areas changed throughout the day due to solar movement, the positions of the weather stations at points A, B, and C were slightly adjusted over time. Point D, located along the visitor pathway, measured thermal conditions in both sunny and shaded environments to assess visitors’ thermal comfort under different conditions. All weather stations recorded data at 1 min intervals.

The Rayman model [37,38] was used to calculate the mean radiant temperature (Tmrt) for the measurement point. The wet-bulb globe temperature (WBGT) [39] was used as the composite thermal environment indicator. WBGT is a composite index commonly employed to assess heat stress in workplaces and sports environments. Unlike other indices frequently used in outdoor thermal comfort research—such as PET [40], UTCI [41], OUT_SET* [42] —WBGT is a purely environmental indicator that does not account for human energy balance. Additionally, WBGT has been used in studies on the thermal environment of livestock and poultry [43,44,45,46], making it well-suited for the purposes of this study. This study used the WBGT calculation following method proposed by [46]. The used formula is given in Equation (1).

(1) $$WBGT=0.735\times Ta+0.0374\times RH+0.00292\times Ta\times RH+7.619\times G-4.557\times G^{2}-0.0572\times Ws-4.064$$

where *Ta* is air temperature (°C), *RH* is relative humidity (%), *G* is global radiation (K·W/m^2^), and *Ws* is wind speed (m/s).

### 2.5. Questionnaire Surveys

**Figure 3:**
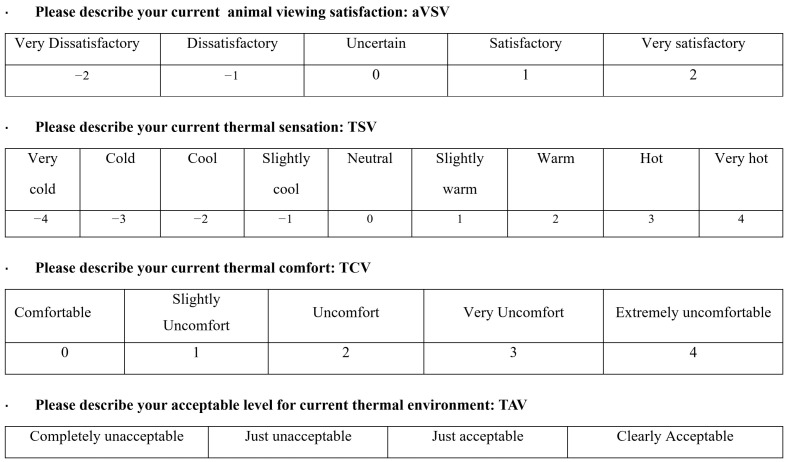
Thermal Perception and Animal Viewing Satisfaction Voting Scales. The thermal sensation survey includes Thermal Sensation Vote (TSV), Thermal Comfort Vote (TCV), and Thermal Acceptability Vote (TAV). According to the ISO 10551 (2019) standard [48], the TSV is measured on a 9-point scale, the TCV on a 5-point scale, and the TAV on a 4-point scale. The figure illustrates the scales used to measure various perceptions in the study. The Thermal Sensation Vote (TSV) ranges from −4 (very cold) to 4 (very hot), the Thermal Comfort Vote (TCV) ranges from 0 (comfortable) to 4 (extremely uncomfortable), and the Thermal Acceptability Vote (TAV) ranges from “clearly acceptable” to “clearly unacceptable”. The Animal Viewing Satisfaction Vote (aVSV) was measured using a 5-point Likert scale, ranging from −2 (very dissatisfactory) to 2 (very satisfactory).

A questionnaire survey was conducted with zoo visitors to gather data relevant to this study. Researchers randomly selected visitors on-site, explained the purpose of the study, and distributed the questionnaires after obtaining informed consent. Each respondent completed one questionnaire. To ensure that the recorded thermal environment data accurately reflected the conditions experienced by respondents, questionnaires were distributed near the meteorological station. The questionnaire comprised three sections. The first section collected respondents’ demographic information, including gender, age, clothing, and activity levels. The second section focused on visitors’ experiences, particularly their animal viewing satisfaction vote (aVSV) and preferences for blue peafowl behaviors. The aVSV was assessed using a 5-point Likert scale [47]. The third part assessed the visitors’ thermal perception. Relevant options for this study included the thermal sensation vote (TSV), thermal comfort vote (TCV), and thermal acceptability vote (TAV) (Figure 3).

### 2.6. Statistical Analysis

This study employed five statistical methods for data analysis. First, the post hoc Tukey test was used to analyze differences in thermal environmental factors across different measurement points. Second, linear and quadratic regression analyses were used to examine the relationships between the comprehensive thermal environment index (WBGT) and visitors’ subjective thermal sensations, visitor numbers, and blue peafowls’ thermal mitigation behaviors. Third, two ordered logistic regression models were developed. The first model included thermal physical factors (air temperature, relative humidity, wind speed, and global radiation) and visitor demographics (gender, clothing, time, and age) as independent variables, with visitor thermal sensation vote (TSV), thermal comfort vote (TCV), thermal acceptability vote (TAV), and aVSV as dependent variables. The second model incorporated the same thermal physical factors, along with blue peafowl gender, visitor feeding behavior, time of visit, and visitor numbers as independent variables, and the proportions of thermal mitigation behaviors (shade-seeking, dynamic activities, feather-spreading, and gular flutter) as dependent variables. These models were designed to assess the impact of various factors on visitors’ thermal perception, viewing satisfaction, and peafowl thermal mitigation behaviors. Finally, to further investigate the influence of thermal physical factors, a random forest model was used to quantify the importance of these features. Unlike ordered logistic regression, the random forest model can capture nonlinear relationships and interactions among these factors.

## 3. Results

### 3.1. Meteorological Parameters

The thermal environment data across measurement points in the zoo was systematically recorded during the survey, revealing variations in temperature, radiation, humidity, and wind speed (Table 3). Point A, located in a direct sunlight area, exhibited the highest average and maximum temperature and radiation values among the four points, with the maximum radiation reaching 1087 W/m^2^, indicating prolonged exposure to intense sunlight. Additionally, the temperature and radiation fluctuate significantly. Point B had the highest average humidity (57.1%), with a maximum value of 90.6%, indicating significant moisture accumulation in the area. Due to its smallest SVF value, the global radiation at Point B was significantly lower than at other points, with an average of only 74 W/m^2^ and a maximum of 188 W/m^2^, suggesting effective shading in the area. Point C had slightly higher global radiation and temperature than Point B, likely due to a lack of surrounding shrubs and slightly higher wind speed. Point D, located on a partially shaded walkway, was divided into D-1 (sunlit) and D-2 (shaded). D-1 exhibited higher global radiation, similar to Point A, with slightly higher temperature and lower humidity compared to D-2. Wind speeds across all points were low, averaging 0.16 to 0.28 m/s, likely due to the dense tree and shrub planting.

### 3.2. Visitors Thermal Comfort and Viewing Satisfaction

#### 3.2.1. Thermal Comfort

**Figure 4:**
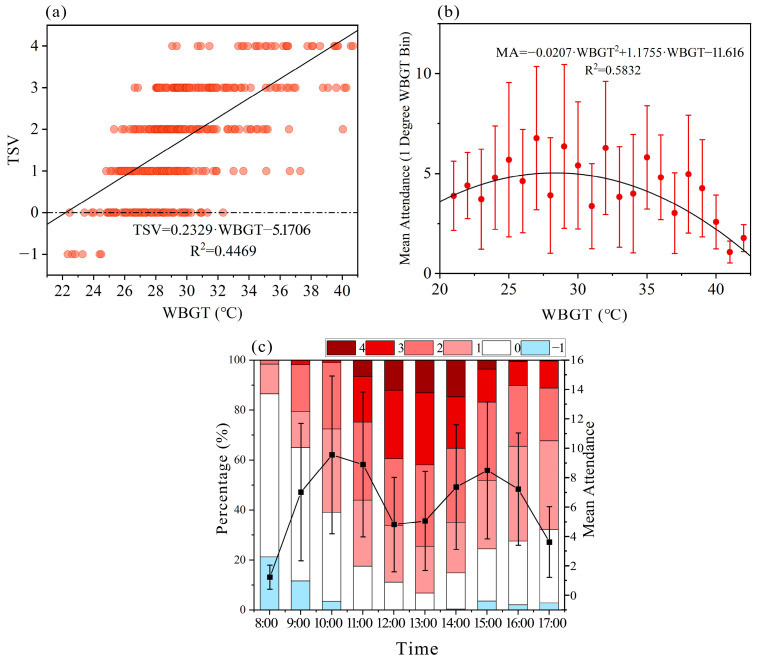
(**a**) Visitor thermal sensation vote (TSV) as a function of Wet-Bulb Globe Temperature (WBGT). (**b**) Average attendance as a function of WBGT. (**c**) Percentage of visitor thermal sensation vote (TSV) and mean attendance over time.

A total of 477 visitor questionnaires were collected. The relationship between visitors’ thermal sensation vote (TSV) and the wet-bulb globe temperature (WBGT) is shown in a scatter plot (Figure 4a) with a linear regression applied. The results indicated that as the thermal environment becomes hotter, TSV increases accordingly. The regression line was segmented: TSV values from −0.5 to 0.5 were categorized as TSV = 0, values from 0.5 to 1.5 as TSV = 1.5, and so on. The corresponding WBGT ranges for each TSV category are: WBGT 20.1–24.4 °C for TSV = 0, 24.4–28.7 °C for TSV = 1, 28.7–32.9 °C for TSV = 2, 32.9–37.2 °C for TSV = 3, and above 37.2 °C for TSV = 4.

Attendance is an important indicator of the success of public spaces, often described as “voting with one’s feet”. Using the same method, the average attendance for each 1 °C WBGT interval was calculated. The change in visitor attendance with WBGT is shown in (Figure 4b). Our results demonstrated that visitor attendance peaked at a WBGT of 28.4 °C, beyond which a gradual decline in numbers was observed. Defining 80% of peak attendance as the threshold for accelerated decline [49], we found visitor numbers decreased sharply when WBGT exceeded 35.4 °C.

The variation in visitor TSV percentage and average attendance throughout the day is shown in (Figure 4c). In the early morning hours, the highest percentage of visitors reported a neutral thermal sensation. As the day progressed, the proportion of visitors with a TSV of zero gradually decreased, while the proportion of votes indicating warmth gradually increased. Between 13:00 and 14:00 h of the day, the votes for thermal sensation levels 3 and 4 reached their peak. After 14:00 h, the proportion of neutral thermal sensation votes slowly began to increase. Visitor attendance peaks twice during the day, with the highest numbers recorded between 09:00–11:00 and 14:00–16:00 h, while the lowest attendance was observed in the early morning hours.

#### 3.2.2. Animal Viewing Satisfaction Vote

**Figure 5:**
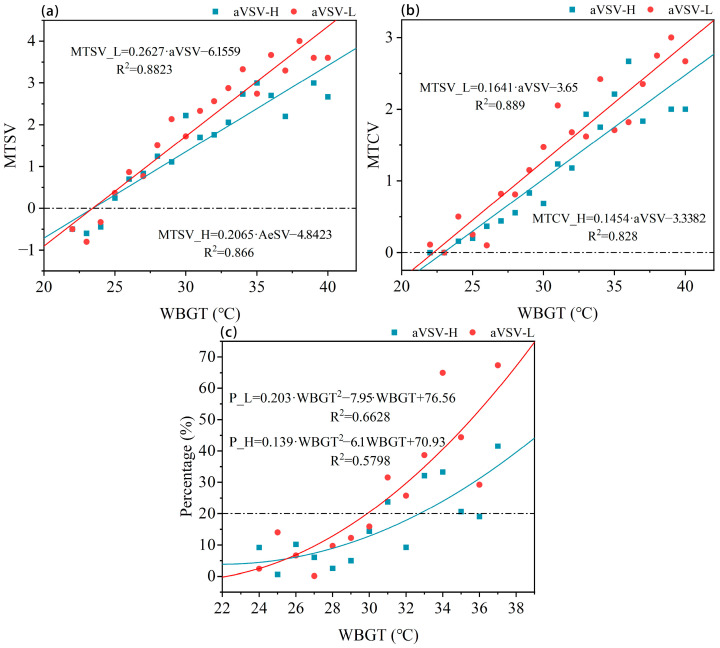
Comparison of thermal perception between high and low aVSV (aVSV-H, aVSV-L) Visitors: (**a**) Mean thermal sensation vote (MTSV) vs. Wet-bulb globe temperature (WBGT). (**b**) Mean Thermal comfort vote (MTCV) vs. WBGT. (**c**) Unacceptability rate of thermal environment and acceptable range.

The animal viewing satisfaction (aVSV) scores were categorized as follows: [−2, 0] for the low satisfaction group (aVSV-L) and [1, 2] for the high satisfaction group (aVSV-H). The scatter distribution of mean thermal sensation vote (MTSV), mean thermal comfort vote (MTCV), and thermal acceptability vote (TAV) with WBGT for aVSV-H and aVSV-L groups within a 1 °C WBGT range is shown in (Figure 5). Linear regression analysis showed that the regression slope for aVSV-H was lower than that for aVSV-L. Near the neutral thermal environment, the difference in TSV between the two groups was minimal. However, environment as WBGT increased, the difference in TSV gradually widened significantly. A similar trend was observed in MTCV scatter points. The regression line for aVSV-H consistently remained lower than that for aVSV-L group, indicating that visitors with higher satisfaction regarding the blue peafowl display experienced greater comfort under the same thermal environment.

The scatter distribution of unacceptable thermal environment rates for satisfied and unsatisfied visitor groups is shown in Figure 5c. The thermal acceptability range for visitors was defined as an unacceptable rate lower than 0.2. For visitors unsatisfied with the peafowl display, the thermal acceptability range was WBGT < 29.8 °C, whereas for satisfied visitors, the acceptable range was WBGT < 32.7 °C.

### 3.3. Heat Adaptation Behaviors of Blue Peafowls

#### 3.3.1. Thermal Mitigation Behaviors and Thermal Environment

**Figure 6:**
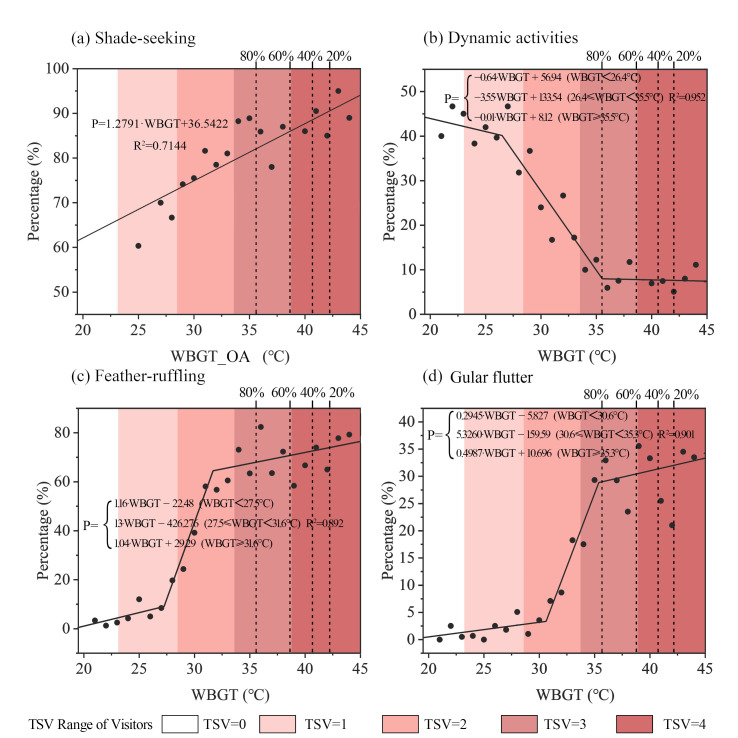
Variation of blue peafowl thermal mitigation behaviors with the thermal environment. (WBGT: Wet-Bulb Globe Temperature; WBGT_OA: WBGT of open areas).

The percentage of blue peafowls performing thermal mitigation behaviors (SS, DA, FS, and GF) was calculated for each 1 °C WBGT interval. The scatter plots of the percentage of blue peafowls exhibiting these behaviors in relation to the thermal environment are shown in Figure 6. For SS analysis, the WBGT of open areas (WBGT_OA) was used as the horizontal axis, while the percentages of DA, FS, and GF were plotted against the WBGT of the specific environment the peafowls occupied. The proportions of SS, FS, and GF increased with rising WBGT, while the proportion of DA decreased as WBGT increased.

A linear regression analysis of the scatter plots revealed that the percentages of DA, FS, and GF remained relatively stable in thermally neutral and high-temperature ranges but changed rapidly during the transitional phase, leading to the adoption of segmented linear regression. The first inflection point in each regression line was identified as the critical WBGT at which blue peafowls initiated thermal mitigation behaviors. According to the regression results, the critical WBGT for reducing DA was 26.4 °C, for starting FS was 27.6 °C, and for initiating GF was 30.4 °C.

Since SS was analyzed using WBGT data from open areas, which lacked data from cooler environments, its scatter plot did not exhibit the same trends as the other thermal mitigation behaviors. Therefore, no inflection point for SS was identified in this study.

The gradient colors in Figure 6 represent the visitors’ Thermal Sensation Votes (TSV), with the top axis indicating the percentage of visitors relative to the maximum attendance. Comparing visitor TSV with the thermal mitigation behaviors of blue peafowls reveals that peafowls performed fewer thermal mitigation behaviors when visitors reported a neutral thermal sensation. When visitors felt “slightly warm”, the peafowls began to reduce DA and started FS. When visitors reported feeling “warm”, the peafowls-initiated GF. This suggests that the thermal comfort ranges of blue peafowls and humans may overlap significantly.

However, the onset of heat stress in peafowls did not coincide with a significant drop in visitor numbers. When visitor attendance declined to 80% of its peak, the blue peafowls had already reduced their DA to very low levels, with the proportion of individuals performing FS and GF reaching a relatively high and stable level to enhance heat dissipation.

#### 3.3.2. Behavior and Time

**Figure 7:**
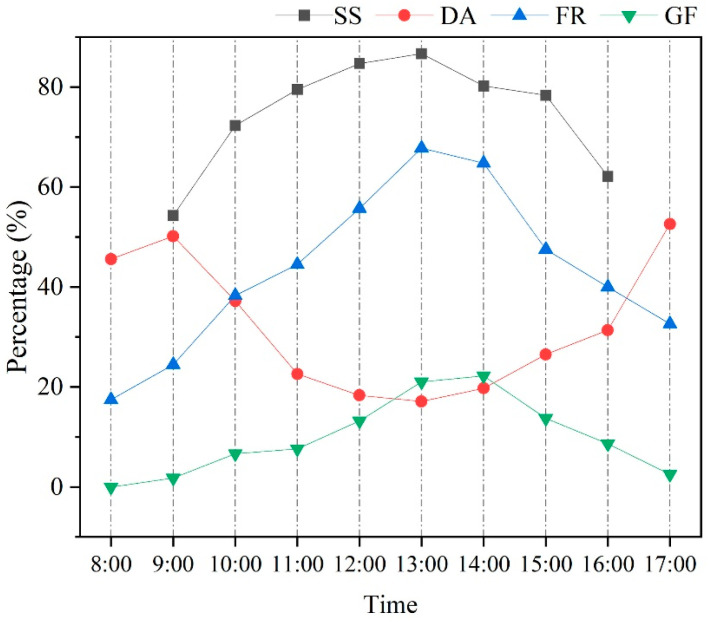
Percentage of blue peafowl thermal mitigation behaviors (dynamic activities (DA), shade-seeking (SS), feather-spreading (FS), and gular flutter (GF)) over time.

The average occurrence rates of thermal mitigation behaviors in blue peafowls throughout the day during the study period are shown in Figure 7. The shed-seeking behaviors (SS) were only recorded between 09:00 and 16:00 h. The occurrence rates of SS, FS, and GF were lowest in the early morning. As the day progressed, the occurrence rates of FS and GF peaked between 13:00 and 14:00 h, then gradually decreased after 15:00 h. The occurrence rate of DA reached its lowest point between 11:00 and 14:00 h (during midday). In contrast, the peak rate of DA occurred later in the afternoon. DA was also relatively high between 8:00 and 9:00 h but did not reach the same levels as observed around 17:00.

### 3.4. Importance of Thermal Environmental Factors

#### 3.4.1. Multi-Factor Analysis

**Figure 8:**
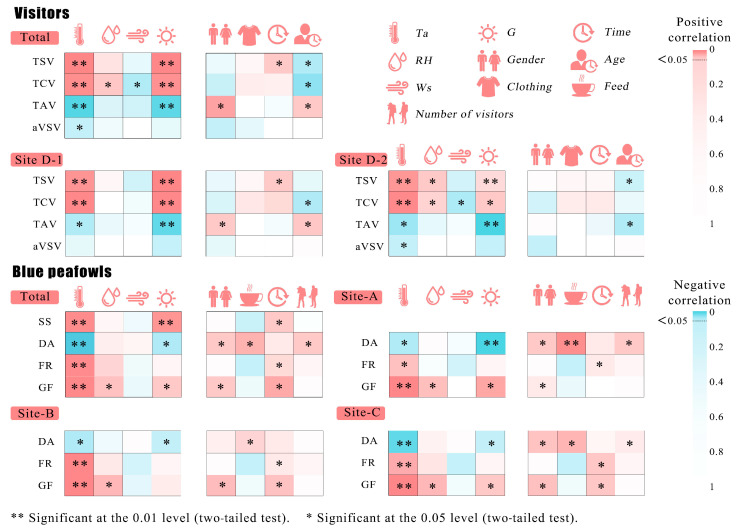
Results of ordinal logistic regression model regression estimates. Correlations between meteorological and social factors and visitors’ Thermal Sensation Vote (TSV), Thermal Comfort Vote (TCV), Thermal Acceptability Vote (TAV), Animal Viewing Satisfaction Vote (aVSV), as well as the frequency of blue peafowls’ dynamic activities (DA), shade-seeking (SS), feather-spreading (FS), and gular flutter (GF). Darker red indicates stronger positive correlations, while darker blue indicates stronger negative correlations.

For visitors, the thermal physical factors air temperature (Ta) and global radiation (G) showed significant correlations with thermal perception votes, including TSV, TCV, and TAV (Figure 8). In shaded environments, RH exhibited a significant positive correlation with TCV, while Ws showed a significant negative correlation with TCV. The overall influence of personal factors on thermal perception was relatively weak; however, under direct sunlight, gender and age significantly affected TCV and TAV. The time of visit had a significant effect on TSV, which may be related to the time-dependent variation of the thermal environment throughout the day. Among the thermal environment factors, Ta showed a significant negative correlation with visitors’ aVSV. Other thermal factors did not show significant correlations with aVSV. Personal factors also did not exhibit significant correlations with aVSV.

Thermal environment factors showed a strong correlation with the thermal mitigation behaviors of blue peafowls. Overall, Ta was significantly correlated with all four types of blue peafowl thermal mitigation behaviors. G showed no significant correlation with FR, while blue peafowl’s GF consistently exhibited a significant positive correlation with RH. Other factors also showed certain correlations with peafowl thermal mitigation behaviors, with feeding always positively correlated with DA. The gender of blue peafowls showed significant correlations with both DA and GF, which may be related to the peafowls’ behavior and physiological structure. Visitor numbers only showed a significant correlation with DA at the open A point and no significant correlation with other blue peafowl thermal mitigation behaviors. This may be because the peafowls had adapted to visitor activity within the zoo, and only at the open A point, where there was more interaction between visitors and peafowls, did it influence the peafowls.

#### 3.4.2. Random Forest

Our results showed that, for visitors’ subjective thermal sensation, solar radiation was the most important thermal environmental factor, followed by air temperature. Relative humidity and wind speed had a weaker influence (Table 4). For blue peafowl’s thermal mitigation behaviors, air temperature was the most significant factor, followed by relative humidity, radiation, and wind speed. For SS and FG, solar radiation was more important than relative humidity. However, for DA and FS, relative humidity had a higher importance than radiation.

## 4. Discussion

### 4.1. Visitors Thermal Comfort

The wet-bulb globe temperature (WBGT) comfort threshold suggests that individuals generally feel comfortable when WBGT is below 26.6 °C [50]. Similarly, for outdoor construction sites in Guangzhou, the ranges for “warm” and “hot” sensations were 28–30.1 °C and 30.1–34.6 °C, respectively [51]. These thresholds for thermal acceptability and equivalent heat stress are lower than those found in this study. This is consistent with previous research on visitor thermal comfort. Research has found that visitors generally have a broader range of thermal comfort and acceptance than residents [28,29,52,53]. This is partly due to visitors’ specific expectations for their destination and partly because they spend more time outdoors, becoming more acclimated to the outdoor thermal environment [29].

This study found that visitor numbers began to decline significantly (below 80% of the peak value) when WBGT exceeded 35.4 °C, a point at which visitors reported feeling “hot”, surpassing the acceptable thermal threshold. This contrasts with findings from Lin [54], Huang et al. [49], and Shooshtarian et al. [55], who observed that resident activity levels were more affected by thermal conditions. Additionally, the analysis of visitor attendance over time in this study suggests that people preferred visiting the zoo between 10:00–11:00 and 14:00–15:00 despite feeling warm during these periods. Conversely, fewer visitors were present between 08:00–09:00 and 16:00–17:00 h, when neutral thermal sensations were more prevalent. This indicates that although the number of zoo visitors is influenced by the thermal environment, other factors, such as the time of day, may have a greater impact.

Under the same level of heat stress, respondents with higher aVSV had lower thermal sensation vote (TSV) and thermal comfort vote (TCV) in the summer. This finding aligns with the research by Yan et al. [56], which showed a correlation between visual satisfaction and thermal perception. Zhang et al. [57] found that aesthetically pleasing environments help reduce thermal discomfort in public settings. In this study, viewing satisfaction with animals had a similar effect as scenic visual satisfaction in previous studies [56], with visitors with higher aVSV also exhibiting a wider thermal acceptance range compared to those with lower aVSV. This could be attributed to two factors. First, visitors with higher aVSV may have a greater interest in animals, enabling them to tolerate greater heat stress. Second, respondents with higher aVSV may have been more cautious when completing the questionnaire and were more inclined to provide positive evaluations of the thermal environment.

### 4.2. Thermal Mitigation Behaviors of Blue Peafowls

Our findings indicate that peafowls’ heat mitigation behaviors are significantly correlated with variations in the thermal environment during summer. As WBGT increases during summer, blue peafowls tend to stay more frequently in shaded areas, and when WBGT exceeds 26.4 °C, they begin to reduce DA. This finding aligns with field studies on other bird species by Williams et al. [58], Ruth et al. [59], and Barrows et al. [27]. Shaded areas reduce radiative heat transfer and provide lower air temperatures, while decreased activity lowers metabolic heat production—common heat stress strategies adopted by many bird species in hot climates. Interestingly, some peafowls still choose to sunbathe under strong solar radiation, likely due to the need for feather maintenance [60], lipid regulation of plumage [61], and parasite removal [62].

Many studies suggest that evaporative cooling is an important mechanism for birds to maintain normal body temperature in high-temperature environments [21,63,64,65,66]. Birds employ two primary physiological mechanisms to increase evaporative cooling during acute heat exposure. The first involves increasing respiratory rate or performing GF to enhance heat dissipation via the respiratory surface [67]. The second mechanism increases the rate of cutaneous evaporation [21,68]. For galliform birds, the bare, featherless areas of the body serve as essential heat dissipation zones [69].

This study found that when WBGT exceeds 27.4 °C, the frequency of FS increases. This allows trapped hot air beneath the feathers to escape, facilitating heat dissipation from the skin. When WBGT rises above 30.6 °C, the likelihood of GF increases. GF accelerates evaporative cooling by increasing respiratory heat loss. However, both GF and FS increase the metabolic cost for the peafowls. This indicates that under such thermal conditions, shade-seeking and reduced activity alone are insufficient to regulate their body heat effectively.

### 4.3. Optimization Design Strategies for the Blue Peafowl Enclosure

The ideal zoo thermal environment should ensure the comfort and enjoyment of visitors while providing a suitable living environment for the animals. In the summer, despite the high temperatures, the number of visitors remains high. The heat stress leads to visitor discomfort and increases the frequency of heat stress behaviors in peafowls. These behaviors, in turn, affect visitor viewing satisfaction and indirectly influence visitor thermal comfort. This negatively impacts the visitor experience and harms animal welfare.

This study found that the acceptable WBGT threshold for visitors is close to the WBGT threshold at which peafowls exhibit heat stress behaviors. However, the analysis of thermal environment feature importance revealed that visitors and blue peafowls have different sensitivities to environmental factors. For visitors, solar radiation is the most important environmental factor affecting thermal comfort in summer, while blue peafowls may be more sensitive to air temperature. Summer thermal environment optimization for peafowl exhibits should have different priorities. Along visitor pathways, the focus should be on reducing solar radiation. In the peafowl living areas, reducing air temperature should be prioritized. On the one hand, tree-shrub-shaded areas had lower temperatures and radiation; on the other hand, peafowls also tended to stay near shrubs. However, it is important to maintain some areas of unshaded space in the peafowl living area, as a small number of peafowls still choose to remain in the sunlight, even under strong solar radiation.

Water is essential in peafowl enclosures during summer for several reasons. It provides evaporative cooling that reduces Ta, creating more comfortable conditions for both peafowls and visitors. Water supports heat adaptation behaviors like FS and GF, which rely on evaporative cooling to lower body temperature. GF particularly increases respiratory water loss. Shallow water bodies in shaded areas help cool peafowls’ feet, which are critical heat dissipation zones. Misting systems are another effective cooling measure, as they offer higher cooling efficiency. Previous studies have shown that misting improves human thermal comfort [70,71,72]. Misting can also keep the peafowls’ feathers moist, thereby enhancing the efficiency of evaporative cooling from their body surfaces.

Outdoor fans are effective cooling interventions. Ordered logistic regression showed a significant correlation between wind speed and visitor TCV in shaded environments. However, random forest classification indicated that wind speed had minimal impact on human comfort and peafowl thermal behaviors, likely due to low wind speeds in densely vegetated areas. Installing fans in visitor rest areas and shaded peafowl habitats could enhance evaporative cooling efficiency for both groups, reducing heatstroke risk during high temperatures.

Peafowls exhibit fewer heat stress behaviors and more dynamic activity during mornings and evenings when visitor numbers are low. In contrast, during the peak visitor periods of 10:00–11:00 and 14:00–15:00, rising temperatures lead to higher heat stress in peafowls. Optimizing the thermal environment in the peafowl enclosure should focus on these two periods. Cooling systems such as fans and misters should be operated strategically based on the time of day to enhance their effectiveness.

### 4.4. Limitations

This study investigated the thermal comfort of visitors and the heat stress behaviors of peafowls in the zoo’s peafowl enclosure during summer. However, as a subtropical species, the thermal mitigation behaviors of peafowls under cold winter conditions also require investigation. Due to space limitations, this study treated both the blue peafowls and the visitors as homogeneous groups. In reality, children and the elderly constitute a significant portion of zoo visitors, and their thermal comfort may differ substantially from that of young and middle-aged adults. Additionally, male and female peafowls may exhibit different heat stress characteristics. Furthermore, this study only examined the influence of thermal environment and time on visitor numbers, but visitor attendance may also be affected by a wide range of complex social factors. Future research should be aimed to address these differences in detail.

## 5. Conclusions

This study explored the impacts of the summer thermal environment at Jinan Zoo on visitors’ comfort and blue peafowl welfare through environmental monitoring, behavioral observations, and questionnaire surveys. Results showed that the visitors maintained high activity levels in moderate warm conditions but began to experience discomfort at higher heat stress levels. Peafowls adapted to heat by avoiding direct sunlight, reducing movement, and engaging in behaviors like feather-spreading and gular flutter, which helped dissipate body heat. Interestingly, visitors with higher viewing satisfaction tended to report greater thermal comfort and tolerate a broader range of thermal conditions. During peak visitor hours, the environment was often warmer. Visitors could tolerate the warmer conditions, but the peafowls displayed higher levels of heat stress. Peafowl heat mitigation behaviors were likely more influenced by air temperature, while visitor discomfort was more closely related to solar radiation. To address the differing thermal needs of visitors and peafowls, shading strategies are essential. Shaded areas with trees and shrubs provided peafowls with space for heat mitigation behaviors. Shading along visitor pathways can help mitigate the effects of solar radiation, the primary factor affecting visitor discomfort. Incorporating water features and cooling systems, such as misting devices and fans, could further enhance thermal comfort for both visitors and peafowls and contribute to a more comfortable and sustainable zoo environment.

## Figures and Tables

**Table 1:** Ethogram of Blue Peafowl Thermal Mitigation Behaviors.

Behavior	Description
Dynamic activities (DA)	Movement behaviors including walking, gliding, tail-fanning, and feeding.
Shade-seeking (SS)	The blue peafowl moves to a shaded area to avoid direct sunlight.
Feather-spreading (FS)	The blue peafowl lifts its feathers, exposing the down feathers underneath, which increases the surface area for heat dissipation and enhances cooling.
Gular flutter (GF)	The blue peafowl opens its beak while rapidly vibrating its throat pouch (the gular sac) to facilitate evaporative cooling.

**Table 2:** Specifications of imaging and meteorological measurement equipment used in this study.

Equipment	Measurement Parameter	Range	Accuracy
Air temperature and humidity recorder	Ta (°C); RH (%)	−40–80 °C; 0–100%RH	±0.1 °C; ±2%RH
Global radiation sensor	G (W/m^2^)	0–1500 W/m^2^	<5%
Wind speed recorder	Ws (m/s)	0–70 m/s	±0.3 m/s
Thermal imaging camera	Ti (°C)	−20–150 °C	±2% of the maximum reading

**Table 3:** Thermal environment data across measurement points in the blue peafowl enclosure and visitor pathway. The table summarizes the minimum, mean, maximum, and standard deviation values for air temperature (Ta), relative humidity (RH), wind speed (WS), and global radiation (G) recorded at four measurement points: Point A (direct sunlight), Point B (shaded with trees and shrubs), Point C (shaded by trees only), and Point D (visitor pathway divided into sunny (D-1) and shaded (D-2) conditions).

	Site	Ta (°C)	RH (%)	WS (m/s)	G (W/m^2^)
Min	A	25.4	39.5	0	62
	B	24.3	48.2	0	24
	C	24.5	43.5	0	30
	D-1	25.3	44.3	0	165
	D-2	24.2	42	0	42
Mean	A	32.1	50.9	0.28	462
	B	30.5	57.1	0.16	74
	C	31.4	52.8	0.2	105
	D-1	32.5	51.2	0.22	373
	D-2	30.7	55.8	0.26	145
Max	A	36.7	80.9	3.3	1087
	B	34	90.6	1.2	188
	C	35.2	85.2	2.8	364
	D-1	35.9	81.5	3.6	1002
	D-2	35.2	88.4	3.1	270
Standard deviation	A	2.83	7.75	0.38	218.7
	B	2.42	7.51	0.21	34.67
	C	2.53	7.22	0.34	76.35
	D-1	2.85	6.38	0.32	183.71
	D-2	2.6	7.16	0.27	62.6

**Table 4:** Importance of Thermal Environmental Factors. Feature importance of four meteorological factors—air temperature (Ta), relative humidity (RH), wind speed (Ws), and global radiation (G)—on visitors’ thermal sensation vote (TSV), thermal comfort vote (TCV), and thermal acceptability vote (TAV), as well as on peafowls’ dynamic activities (DA), shade-seeking (SS), feather-spreading (FS), and gular flutter (GF).

		Ta	RH	Ws	G
Visitors	TSV	0.234	0.175	0.08	0.511
	TCV	0.339	0.102	0.133	0.426
	TAV	0.265	0.224	0.101	0.41
Thermal mitigation Behaviors of Blue Peafowls	SS	0.47	0.202	0.025	0.302
	DA	0.63	0.183	0.009	0.178
	FS	0.538	0.232	0.089	0.141
	GF	0.601	0.227	0.01	0.163

## Data Availability

The data presented in this study are available on request from the corresponding author.

## Abbreviations

aVSV	Animal viewing satisfaction vote
TSV	Thermal sensation vote
TAV	Thermal acceptability vote
Ta	Air temperature
Ws	Wind speed
RH	Relative humidity
G	Global radiation
SVF	The sky view factors
Tmrt	Mean radiant temperature
WBGT	Wet-bulb globe temperature
WBGT_OA	WBGT of open areas
DA	Dynamic activities
SS	Shade-seeking
FS	Feather-spreading
GF	Gular flutter
Tmrt_OA	Mean radiant temperature of the open area
